# Synthesis, crystal structure and Hirshfeld surface analysis of 2-[(4-hy­droxy­phen­yl)amino]-5,5-diphenyl-1*H*-imidazol-4(5*H*)-one

**DOI:** 10.1107/S2056989024003499

**Published:** 2024-04-26

**Authors:** Abderrazzak El Moutaouakil Ala Allah, Walid Guerrab, Joel T. Mague, Abdulsalam Alsubari, Abdullah Yahya Abdullah Alzahrani, Youssef Ramli

**Affiliations:** a Laboratory of Medicinal Chemistry, Drug Sciences Research Center, Faculty of Medicine and Pharmacy, Mohammed V University in Rabat, Morocco; b Department of Chemistry, Tulane University, New Orleans, LA, 70118, USA; c Laboratory of Medicinal Chemistry, Faculty of Clinical Pharmacy, 21 September University, Yemen; d Department of Chemistry, Faculty of Science and Arts, King Khalid University, Mohail Assir, Saudi Arabia; Katholieke Universiteit Leuven, Belgium

**Keywords:** crystal structure, hydrogen bond, C—H⋯π(ring) inter­action, amine, di­hydro­imidazolone, hydantoin

## Abstract

In the title mol­ecule, C_21_H_17_N_3_O_2_, the five-membered ring is slightly ruffled and dihedral angles between the pendant six-membered rings and the central, five-membered ring vary between 50.78 (4) and 86.78 (10)°. The exocyclic nitro­gen lone pair is involved in conjugated π bonding to the five-membered ring. In the crystal, a layered structure is generated by O—H⋯N and N—H⋯O hydrogen bonds plus C—H⋯π(ring) and weak π-stacking inter­actions.

## Abstract 2

The five-membered ring is slightly ruffled and dihedral angles between the pendant six-membered rings and the central, five-membered ring vary between 50.78 (4) and 86.78 (10)°. In the crystal, O—H⋯N hydrogen bonds and weak π-stacking inter­actions form inversion dimers, which are linked into chains extending along the *c*-axis direction by two sets of N—H⋯O hydrogen bonds. The chains are connected into layers parallel to the *bc* plane by two sets of C—H⋯π(ring) inter­actions.

## 1. Chemical context

Hydantoins or imidazolidine-2,4-diones are heterocyclic compounds characterized by the presence of an imidazole ring and keto groups in positions 2 and 4. Hydantoin-containing compounds exhibit a broad spectrum of pharmacological and biological activities such as an anti­cancer (Cao *et al.*, 2022 ▸), anti­bacterial (Ghasempour *et al.*, 2021 ▸; El Moutaouakil Ala Allah et *al*., 2024 ▸), anti­diabetic (Sergent *et al.*, 2008 ▸), anti-inflammatory (Lin *et al.*, 2021 ▸), anti­microbial (Shaala & Youssef, 2021 ▸), anti­convulsant (Byrtus *et al.*, 2011 ▸) and anti-HIV (Romine *et al.*, 2011 ▸) activities. Thio­hydantoins, sulfur analogues of hydantoins, undergo replacement of one or both carbonyl groups with thio­carbonyl groups (Johnson & Scott, 1913 ▸; Wyzlic *et al.*, 1996 ▸; Cromwell & Stark, 1969 ▸). This substitution enables versatile structural modifications, facilitating the customization of thio­hydantoins to preferentially adopt specific structural types. Such modifications, achieved by introducing steric bulk, altering hydro­philic or hydro­phobic inter­actions, or promoting π–π stacking, afford control over the mol­ecule’s ability to form hydrogen-bonded arrays in the solid state. Hence, the capacity to manipulate the formation of hydrogen-bonded arrays in the solid state is of vital importance in the pharmaceutical field (Lu & Rohani, 2009 ▸). 
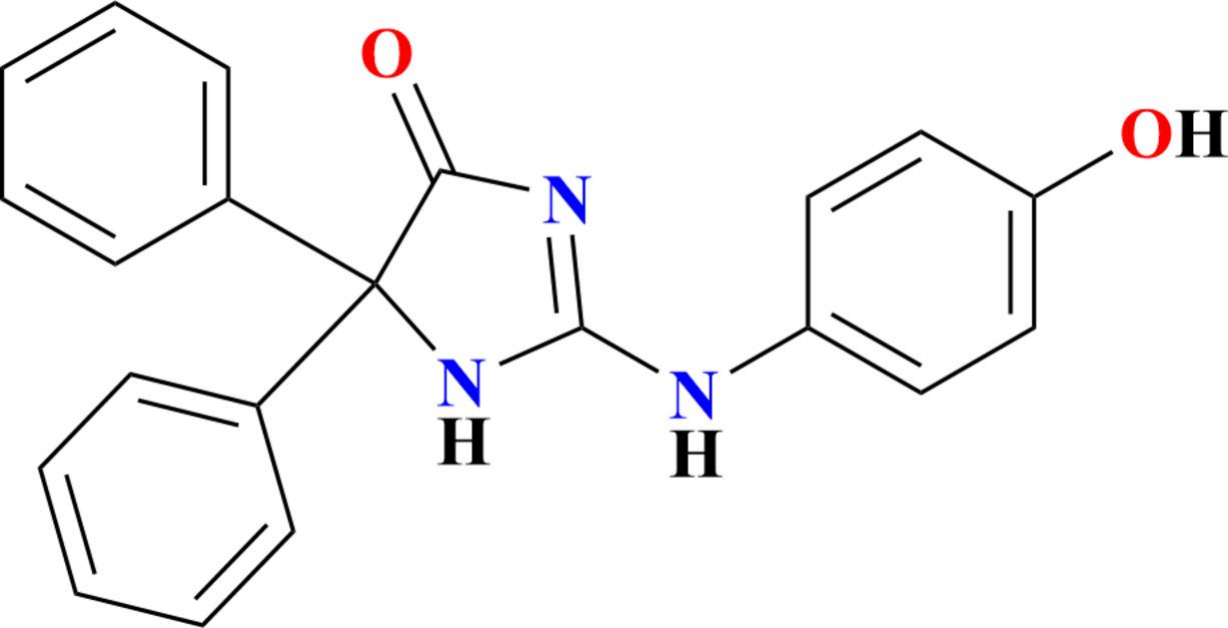


**Figure 1:**
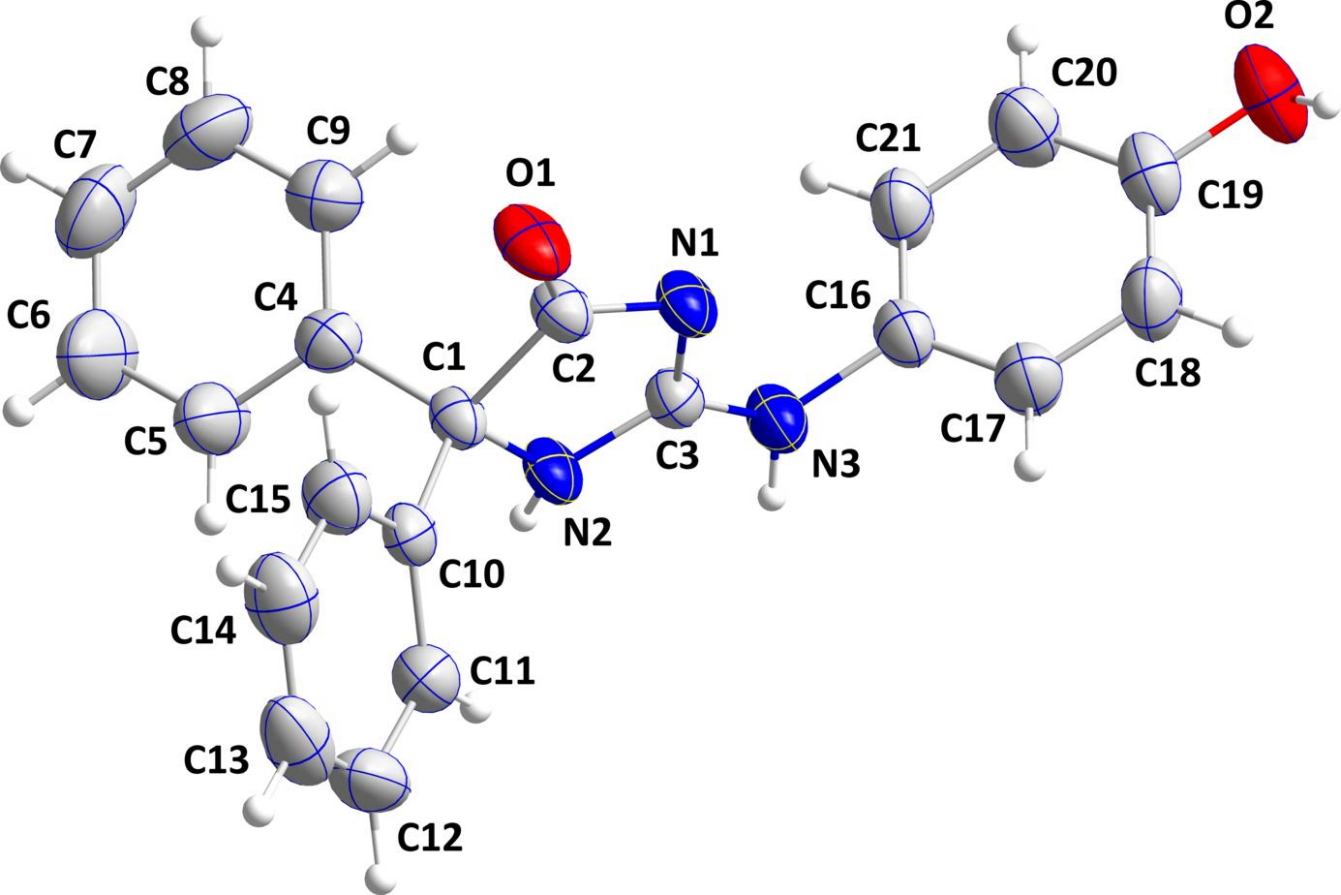
The mol­ecular structure of the title mol­ecule with labelling scheme and 50% probability ellipsoids.

In this study, we present the synthesis, detailed examination of the mol­ecular and crystal structures, and Hirshfeld surface analysis of the title compound, 2-[(4-hy­droxy­phen­yl)amino]-5,5-diphenyl-1*H*-imidazol-4(5*H*)-one (Fig. 1 ▸), a new hydantoin derived from thio­hydantoin by a nucleophilic substitution reaction.

## 2. Structural commentary

The mean planes of the C4–C9, C10–C15 and C16–C21 benzene rings are inclined to that of the C1/C2/N1/C3/N2 ring by 73.33 (9), 50.78 (11) and 86.78 (10)°, respectively. The C16—N3—C3—N1 torsion angle is −7.2 (5)° indicating that the N3—C16 bond lies close to the plane of the C1/C2/N1/C3/N2 ring. This latter ring is slightly ruffled with N2 0.031 (2) Å at one side of the mean plane (r.m.s. deviation of the fitted atoms = 0.002 Å) and C1 0.027 (3) Å at the opposite side. The sum of the angles around N3 is 359.4 (13)° implying that its lone pair is involved in N→C π bonding. This occurs primarily with C3 as the C3—N3 distance is 1.329 (3) Å while the C16—N3 distance is 1.439 (3) Å indicating some degree of conjugation with the di­hydro­imidazolone ring.

## 3. Supra­molecular features

**Figure 2:**
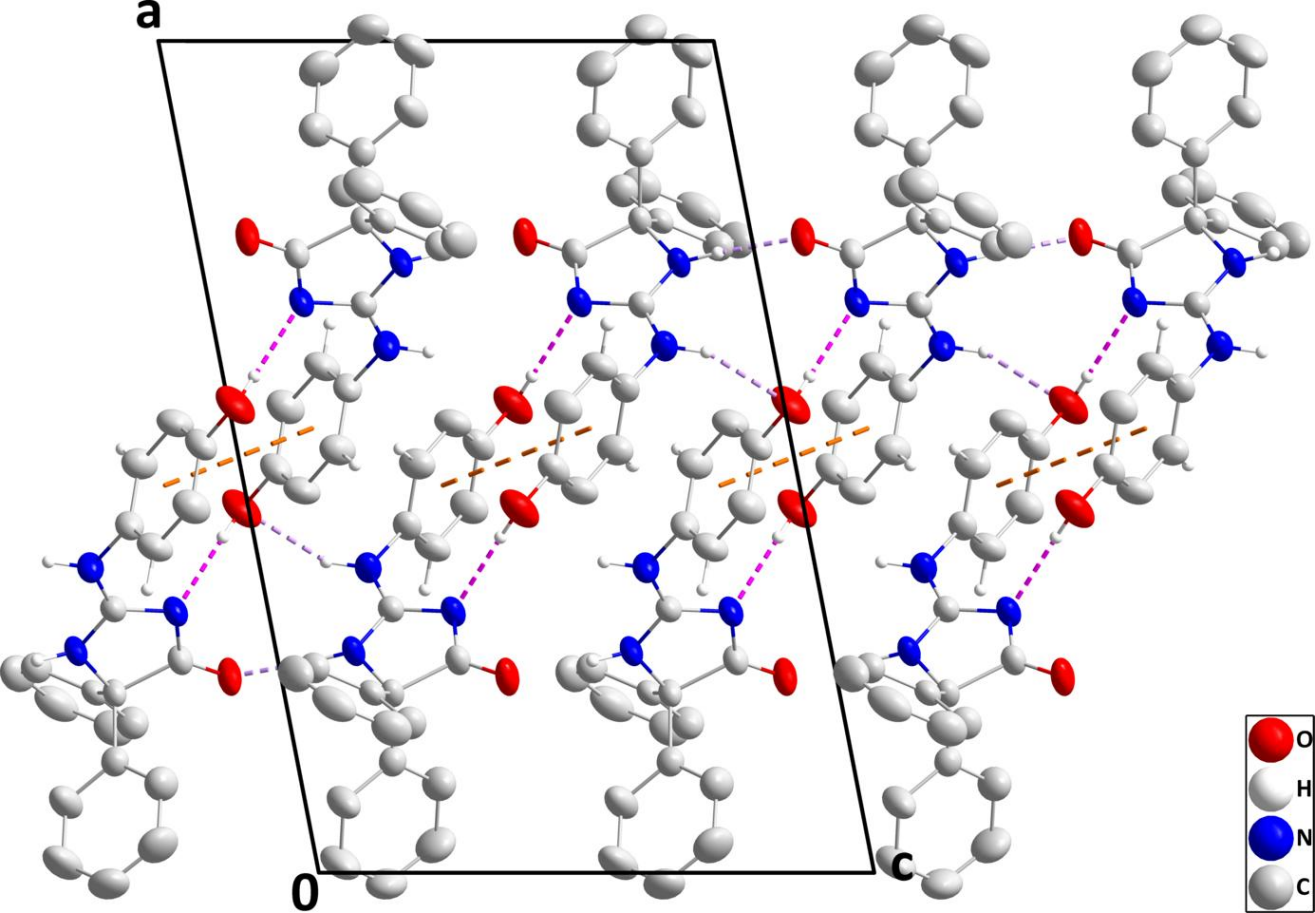
A portion of one chain of mol­ecules viewed along the *b*-axis direction. O—H⋯N and N—H⋯O hydrogen bonds are depicted, respectively, by pink and violet dashed lines and non-inter­acting hydrogen atoms are omitted for clarity.

**Figure 3:**
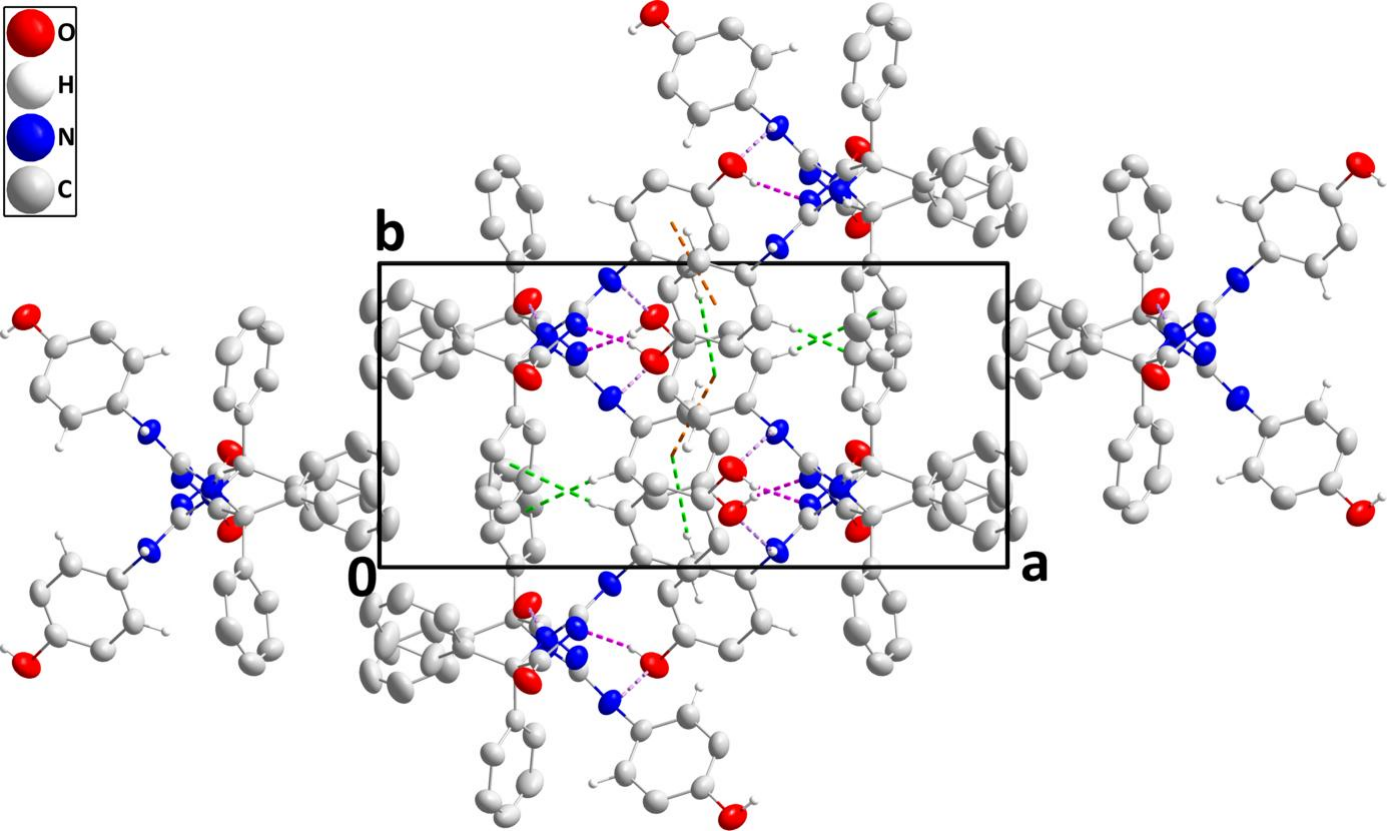
Packing viewed along the *c*-axis direction with inter­molecular hydrogen bonds depicted as in Fig. 2 ▸. C—H⋯π(ring) inter­actions are depicted by green dashed lines and non-inter­acting hydrogen atoms are omitted for clarity.

In the crystal, paired O2—H2*A*⋯N1 hydrogen bonds (Table 1 ▸) and weak, offset π-stacking inter­actions between C16–C21 rings [centroid–centroid distance = 3.9814 (19) Å, offset = 2.23 Å] form inversion dimers, which are connected into chains extending along the *c*-axis direction by N2—H2⋯O1 and N3—H3⋯O2 hydrogen bonds (Table 1 ▸ and Fig. 2 ▸). These are linked into layers parallel to the *bc* plane by C17—H17⋯*Cg*4 and C21—H21⋯*Cg*3 inter­actions (Table 1 ▸ and Fig. 3 ▸; *Cg*3 and *Cg*4 are the centroids of the C10–C15 and C16–C21 benzene rings, respectively).

## 4. Database survey

**Figure 4:**
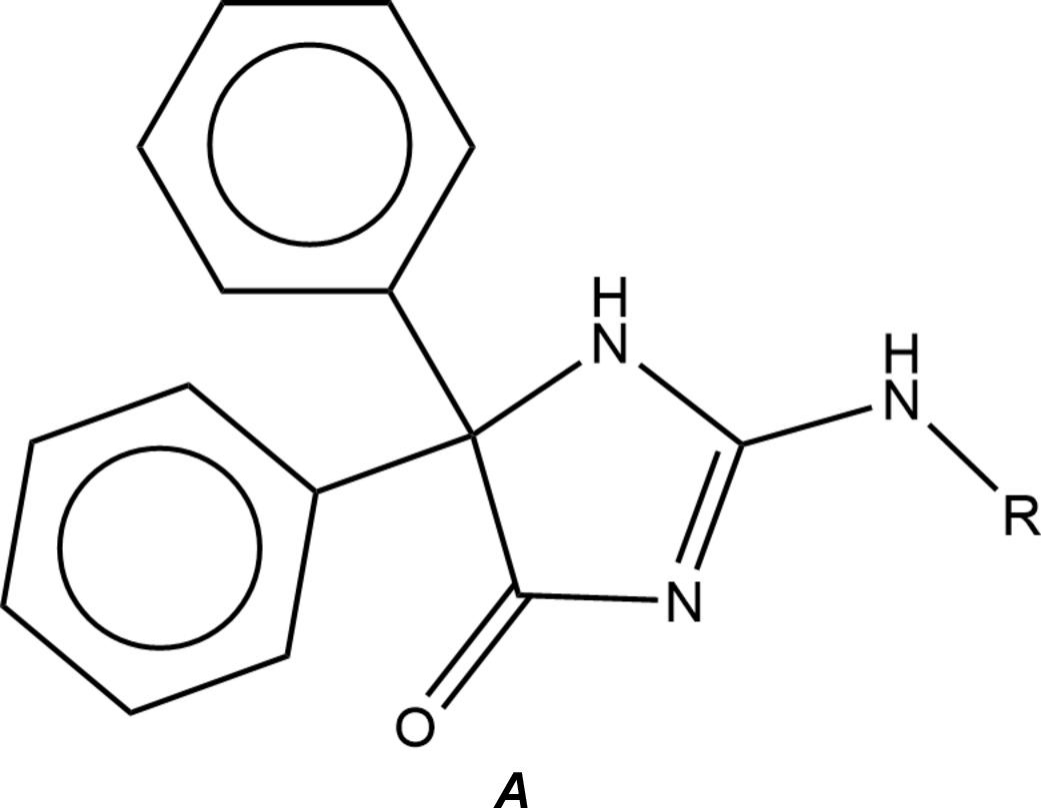
Fragment used in the CSD search.

A search of the Cambridge Structural Database (CSD version 5.45, updated to March 2024; Groom *et al.*, 2016 ▸) with the search fragment *A* (Fig. 4 ▸, *R* = C) gave three hits, one with *R* = CH_2_COOEt (refcode REFREB; Karolak-Wojciechowska *et al.*, 1998 ▸) and the others with *R* = C(=NH)OMe (XASGOO; Bishop *et al.*, 2005 ▸) and *R* = C(=NH)OBu^*n*^ (XEVZEE; Bishop *et al.*, 2007 ▸). The latter two were reported as complexes with Cu^II^ and so are not directly comparable to the title mol­ecule because of the constraints imposed by coordination to the metal. In REFREB, the five-membered ring adopts an envelope conformation with C4 at the tip of the flap and 0.044 (6) Å from the mean plane (r.m.s. deviation of the fitted atoms = 0.003 Å) with the mean planes of the attached phenyl rings inclined to the above plane by 63.3 (2) and 82.9 (2)°, respectively, which are similar to the corresponding angles in the title mol­ecule. Also, the torsion angle corresponding to the C16—N3—C3—N1 angle in the title mol­ecule is for REFREB −8.0 (5)°, which is again comparable to that cited above although the remainder of the ester chain is pointed away from the plane of the five-membered ring.

## 5. Hirshfeld surface analysis

**Figure 5:**
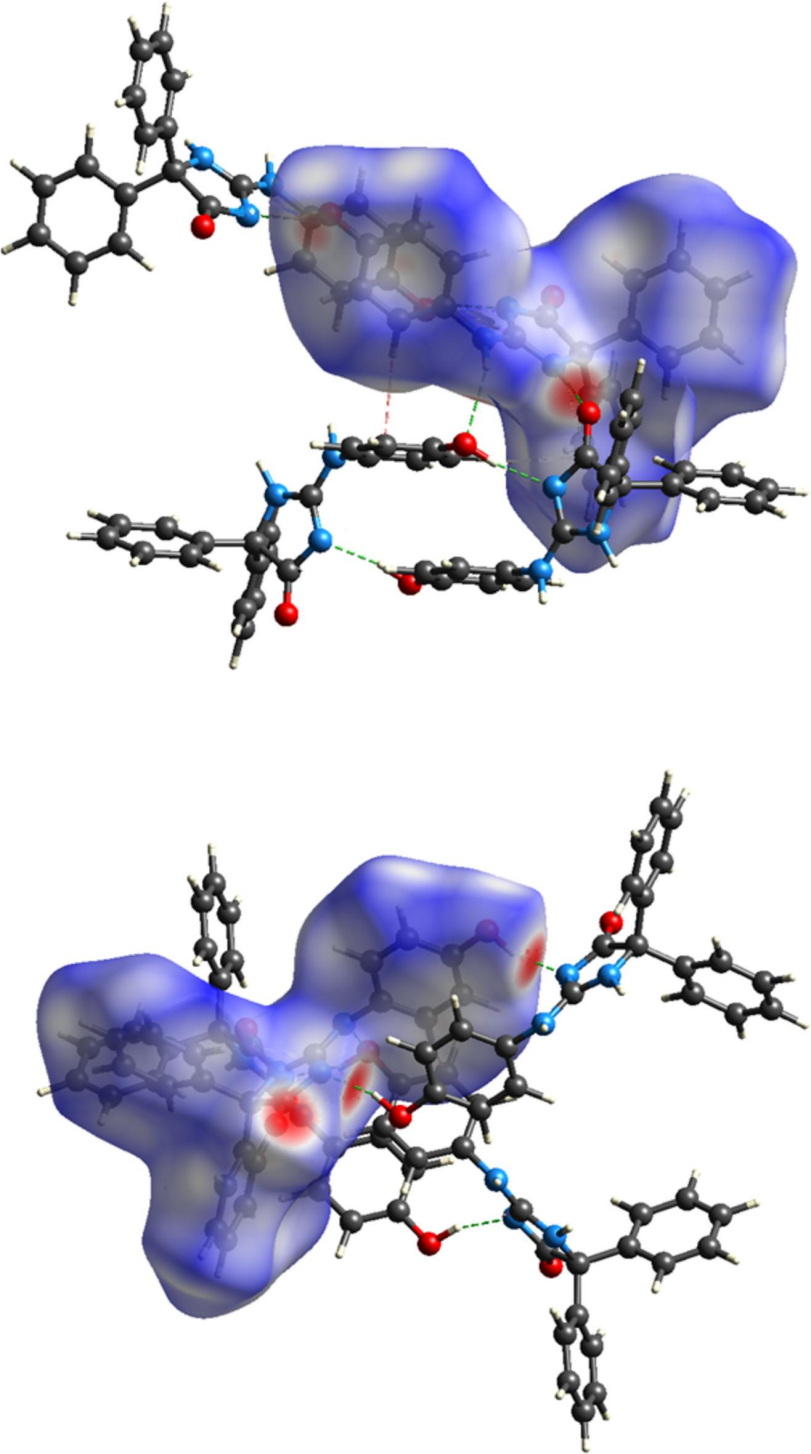
Front and back views of the Hirshfeld surface for the title mol­ecule mapped over *d*_norm_.

**Figure 6:**
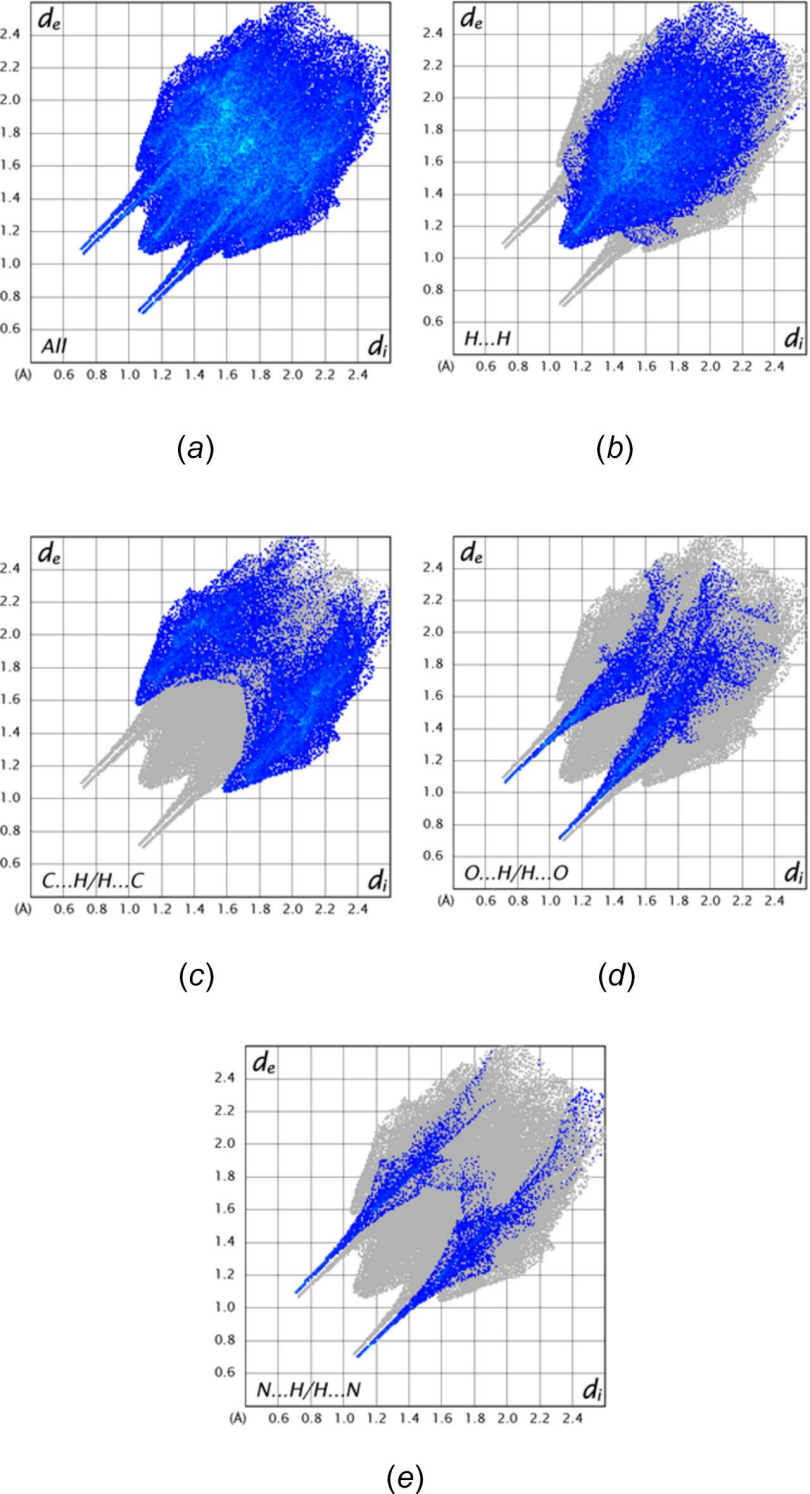
The 2-D fingerprint plots for the title mol­ecule; (*a*) all inter­actions and delineated into (*b*) H⋯H, (*c*) C⋯H/H⋯C, (*d*) O⋯H/H⋯O and (*e*) N⋯H/H⋯N contacts.

A Hirshfeld surface analysis was performed using *CrystalExplorer21* (Turner *et al.*, 2017 ▸) to evaluate the relative contributions of the inter­molecular inter­actions in the crystal. Additional details of the plots produced and their inter­pretation have been published (Tan *et al.*, 2019 ▸). Fig. 5 ▸ presents two views of the surface mapped over *d*_norm_ together with four neighbouring mol­ecules showing the inter­molecular N—H⋯O and O—H⋯N hydrogen bonds as well as one of the C—H⋯π(ring) inter­actions. From the 2D fingerprint plots, the major inter­molecular inter­actions, comprising 48.7% of the total, are H⋯H contacts (Fig. 6 ▸ *b*), appearing as a broad central peak and which are presumed to be van der Waals contacts. At 28.9% of the total are the C⋯H/H⋯C contacts (Fig. 6 ▸ *c*), shown as two broad peaks at *d*_e_ + *d*_i_ = 3.14 Å, which are primarily the two sets of C—H⋯π(ring) inter­actions (Table 1 ▸) with the width of the peaks due to the range of H⋯C distances from the hydrogen atom in question to the several carbon atoms of the ring. The O⋯H/H⋯O (Fig. 6 ▸ *d*) and N⋯H/H⋯N (Fig. 6 ▸ *e*) contacts appear as sharp spikes at *d*_e_ + *d*_i_ = 2.16 and 2.20 Å, respectively, contributing 13.3% and 6.9%, respectively.

## 6. Synthesis and crystallization

**Figure 7:**
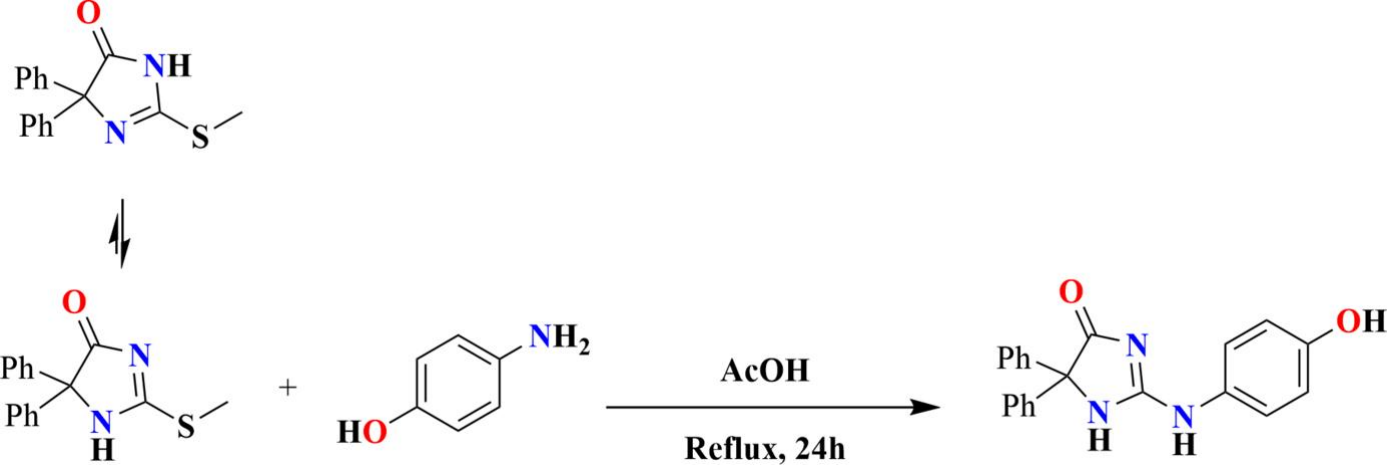
Synthesis of the title compound.

The synthesis of the title compound is shown in Fig. 7 ▸. 2-(Methyl­thio)-5,5-diphenyl-3,5-di­hydro-4*H*-imidazol-4-one (0.5 g, 1.78 mmol) and 4-amino­phenol (0.2 g, 1.80 mmol) were dissolved in 30 ml of glacial acetic acid. The reaction mixture was heated under reflux for 24 h and the reaction progress was monitored with thin-layer chromatography (TLC). The precipitated solid was filtered, washed with water, dried and purified by recrystallization from ethanol to afford colourless crystals.

Yield = 68%, m.p. = 424-425 K. FT–IR (ATR, υ, cm^−1^): 3385 (OH), 3200 (NH), 1740 (C=O); ^1^H NMR (500 MHz, CDCl_3_): δ ppm 7.26–7.62 (*m*, 14H, Ar-**H**), 9.17 (*s*, 1H, N**H**_imidazole_), 9.95 (*s*, 1H, N**H**_amine_), 10.11 (*s*, 1H, O**H**); ^13^C NMR: 78.53 (**C**-2Ph); 116.00, 116.18, 123.89, 127.62, 128.02, 128.74, 130.57, 135.00 (**C–**-Ar); 141.36 (**C**=N); 168.32 (**C**=O). HRMS (ESI): calculated for C_21_H_17_N_3_O_2_ [*M* - H]^+^ 344.1321; found 344.1520.

## 7. Refinement

Crystal data, data collection and structure refinement details are summarized in Table 2 ▸. Analysis of 185 reflections having *I*/σ(*I*) > 12 and chosen from the full data set with *CELL_NOW* (Sheldrick, 2008*a* ▸) showed the crystal to belong to the monoclinic system and to be twinned by a 180° rotation about the c*-axis. The raw data were processed using the multi-component version of *SAINT* under control of the two-component orientation file generated by *CELL_NOW*. The final refinement used the full twinned dataset. H atoms attached to carbon were placed in calculated positions and were included as riding contributions with isotropic displacement parameters 1.2–1.5 times those of the attached atoms. Those attached to nitro­gen and to oxygen were placed in locations derived from a difference map and refined with DFIX 0.91 0.01 and DFIX 0.84 0.01 instructions, respectively. One reflection affected by the beamstop was omitted from the final refinement.

## Supplementary Material

Crystal structure: contains datablock(s) global, I. DOI: 10.1107/S2056989024003499/vm2300sup1.cif

Structure factors: contains datablock(s) I. DOI: 10.1107/S2056989024003499/vm2300Isup2.hkl

Supporting information file. DOI: 10.1107/S2056989024003499/vm2300Isup3.cml

CCDC reference: 2349453

Additional supporting information:  crystallographic information; 3D view; checkCIF report

## Figures and Tables

**Table 1:** Hydrogen-bond geometry (Å, °) *Cg*3 and *Cg*4 are the centroids of the C10–C15 and the C16–C21 benzene rings, respectively.

*D*—H⋯*A*	*D*—H	H⋯*A*	*D*⋯*A*	*D*—H⋯*A*
O2—H2*A*⋯N1^i^	0.86 (1)	1.93 (2)	2.763 (3)	163 (4)
N2—H2⋯O1^ii^	0.90 (1)	1.92 (1)	2.814 (3)	176 (3)
N3—H3⋯O2^iii^	0.89 (1)	2.34 (2)	3.104 (4)	143 (2)
C17—H17⋯*Cg*4^iii^	0.95	2.92	3.831 (4)	162
C21—H21⋯*Cg*3^iv^	0.95	2.93	3.822 (4)	157

Symmetry codes: (i) 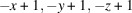 ; (ii) 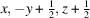 ; (iii) 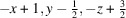 ; (iv) 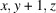 .

**Table 2:** Experimental details

Crystal data	
Chemical formula	C_21_H_17_N_3_O_2_
*M*_r_	343.38
Crystal system, space group	Monoclinic, *P*2_1_/*c*
Temperature (K)	150
*a*, *b*, *c* (Å)	17.764 (3), 8.4429 (12), 11.6601 (16)
β (°)	100.948 (4)
*V* (Å^3^)	1716.9 (4)
*Z*	4
Radiation type	Mo *K*α
μ (mm^−1^)	0.09
Crystal size (mm)	0.38 × 0.21 × 0.02

Data collection	
Diffractometer	Bruker D8 QUEST PHOTON 3 diffractometer
Absorption correction	Multi-scan (*TWINABS*; Sheldrick, 2009 ▸)
*T*_min_, *T*_max_	0.97, 1.00
No. of measured, independent and observed [*I* > 2σ(*I*)] reflections	16776, 5347, 3329
*R*_int_	0.045
(sin θ/λ)_max_ (Å^−1^)	0.627

Refinement	
*R*[*F* ^2^ > 2σ(*F* ^2^)], *wR*(*F* ^2^), *S*	0.055, 0.132, 1.04
No. of reflections	5347
No. of parameters	248
No. of restraints	3
H-atom treatment	H atoms treated by a mixture of independent and constrained refinement
Δρ_max_, Δρ_min_ (e Å^−3^)	0.23, −0.22

Computer programs: *APEX4* and *SAINT* (Bruker, 2021 ▸), *SHELXT* (Sheldrick, 2015*a* ▸), *SHELXL2018/1* (Sheldrick, 2015*b* ▸), *DIAMOND* (Brandenburg & Putz, 2012 ▸) and *SHELXTL* (Sheldrick, 2008*b* ▸).

## supplementary crystallographic information

### Crystal data

**Table:**

C_21_H_17_N_3_O_2_	*F*(000) = 720
*M**_r_* = 343.38	*D*_x_ = 1.328 Mg m^−^^3^
Monoclinic, *P*2_1_/*c*	Mo *K*α radiation, λ = 0.71073 Å
*a* = 17.764 (3) Å	Cell parameters from 3889 reflections
*b* = 8.4429 (12) Å	θ = 2.3–26.2°
*c* = 11.6601 (16) Å	µ = 0.09 mm^−^^1^
β = 100.948 (4)°	*T* = 150 K
*V* = 1716.9 (4) Å^3^	Plate, colourless
*Z* = 4	0.38 × 0.21 × 0.02 mm

### Data collection

**Table:**

Bruker D8 QUEST PHOTON 3 diffractometer	5347 independent reflections
Radiation source: fine-focus sealed tube	3329 reflections with *I* > 2σ(*I*)
Graphite monochromator	*R*_int_ = 0.045
Detector resolution: 7.3910 pixels mm^-1^	θ_max_ = 26.5°, θ_min_ = 2.3°
ω scans	*h* = −22→21
Absorption correction: multi-scan (*TWINABS*; Sheldrick, 2009)	*k* = 0→10
*T*_min_ = 0.97, *T*_max_ = 1.00	*l* = 0→14
16776 measured reflections	

### Refinement

**Table:**

Refinement on *F*^2^	Primary atom site location: dual
Least-squares matrix: full	Secondary atom site location: difference Fourier map
*R*[*F*^2^ > 2σ(*F*^2^)] = 0.055	Hydrogen site location: mixed
*wR*(*F*^2^) = 0.132	H atoms treated by a mixture of independent and constrained refinement
*S* = 1.03	*w* = 1/[σ^2^(*F*_o_^2^) + (0.0457*P*)^2^ + 0.5709*P*] where *P* = (*F*_o_^2^ + 2*F*_c_^2^)/3
5347 reflections	(Δ/σ)_max_ < 0.001
248 parameters	Δρ_max_ = 0.23 e Å^−^^3^
3 restraints	Δρ_min_ = −0.22 e Å^−^^3^

### Special details

**Table:**

Experimental. The diffraction data were collected in three sets of 363 frames 0.5° width in ω) at φ = 0, 120 and 240°. A scan time of 60 sec/frame was used. Analysis of 185 reflections having I/σ(I) > 12 and chosen from the full data set with *CELL_NOW* (Sheldrick, 2008) showed the crystal to belong to the monoclinic system and to be twinned by a 180° rotation about the *c** axis. The raw data were processed using the multi-component version of *SAINT* under control of the two-component orientation file generated by *CELL_NOW*.
Geometry. All esds (except the esd in the dihedral angle between two l.s. planes) are estimated using the full covariance matrix. The cell esds are taken into account individually in the estimation of esds in distances, angles and torsion angles; correlations between esds in cell parameters are only used when they are defined by crystal symmetry. An approximate (isotropic) treatment of cell esds is used for estimating esds involving l.s. planes.
Refinement. Refinement of F^2^ against ALL reflections. The weighted R-factor wR and goodness of fit S are based on F^2^, conventional R-factors R are based on F, with F set to zero for negative F^2^. The threshold expression of F^2^ > 2sigma(F^2^) is used only for calculating R-factors(gt) etc. and is not relevant to the choice of reflections for refinement. R-factors based on F^2^ are statistically about twice as large as those based on F, and R- factors based on ALL data will be even larger. H-atoms attached to carbon were placed in calculated positions (C—H = 0.95 - 0.98 Å) and were included as riding contributions with isotropic displacement parameters 1.2 - 1.5 times those of the attached atoms. Those attached to nitrogen and to oxygen were placed in locations derived from a difference map and refined with DFIX 0.91 0.01 and DFIX 0.84 0.01 instructions, respectively. Refined as a 2-component twin. One reflection affected by the beamstop was omitted from the final refinement.

### Fractional atomic coordinates and isotropic or equivalent isotropic displacement parameters (Å^2^)

**Table:**

	*x*	*y*	*z*	*U*_iso_*/*U*_eq_	
O1	0.76440 (12)	0.1256 (2)	0.59260 (16)	0.0529 (6)	
O2	0.43772 (15)	0.8230 (3)	0.4878 (2)	0.0664 (7)	
H2A	0.4033 (16)	0.767 (4)	0.444 (3)	0.092 (14)*	
N1	0.68601 (13)	0.2980 (3)	0.66727 (18)	0.0409 (6)	
N2	0.73357 (13)	0.2658 (3)	0.86027 (18)	0.0386 (6)	
H2	0.7453 (16)	0.303 (3)	0.9339 (13)	0.047 (9)*	
N3	0.63328 (14)	0.4450 (3)	0.8052 (2)	0.0440 (6)	
H3	0.6262 (16)	0.448 (3)	0.8788 (13)	0.057 (10)*	
C1	0.78406 (15)	0.1675 (3)	0.8050 (2)	0.0342 (6)	
C2	0.74437 (17)	0.1927 (3)	0.6755 (2)	0.0385 (7)	
C3	0.68300 (16)	0.3401 (3)	0.7783 (2)	0.0368 (7)	
C4	0.86637 (16)	0.2335 (3)	0.8283 (2)	0.0394 (7)	
C5	0.91538 (19)	0.1953 (5)	0.9308 (3)	0.0669 (11)	
H5	0.898781	0.124191	0.984000	0.080*	
C6	0.9881 (2)	0.2584 (5)	0.9574 (3)	0.0864 (13)	
H6	1.020691	0.231765	1.029192	0.104*	
C7	1.0135 (2)	0.3585 (5)	0.8820 (4)	0.0766 (12)	
H7	1.063708	0.401801	0.900559	0.092*	
C8	0.9667 (2)	0.3961 (5)	0.7801 (4)	0.0749 (11)	
H8	0.984311	0.465610	0.726783	0.090*	
C9	0.8931 (2)	0.3340 (4)	0.7526 (3)	0.0610 (9)	
H9	0.860949	0.361313	0.680653	0.073*	
C10	0.78376 (15)	−0.0048 (3)	0.8413 (2)	0.0366 (7)	
C11	0.75374 (17)	−0.0543 (4)	0.9371 (3)	0.0491 (8)	
H11	0.728488	0.019425	0.978410	0.059*	
C12	0.7607 (2)	−0.2115 (4)	0.9723 (3)	0.0657 (10)	
H12	0.740132	−0.244907	1.037771	0.079*	
C13	0.7969 (2)	−0.3190 (4)	0.9136 (4)	0.0671 (11)	
H13	0.801641	−0.426344	0.938423	0.081*	
C14	0.8263 (2)	−0.2712 (4)	0.8188 (3)	0.0626 (9)	
H14	0.851184	−0.345553	0.777377	0.075*	
C15	0.81995 (18)	−0.1159 (4)	0.7836 (3)	0.0499 (8)	
H15	0.840871	−0.084081	0.718038	0.060*	
C16	0.58283 (16)	0.5387 (3)	0.7202 (2)	0.0410 (7)	
C17	0.50842 (17)	0.4918 (4)	0.6807 (3)	0.0538 (8)	
H17	0.490481	0.394955	0.707081	0.065*	
C18	0.45952 (18)	0.5859 (4)	0.6024 (3)	0.0558 (9)	
H18	0.407891	0.553935	0.575721	0.067*	
C19	0.48536 (18)	0.7249 (4)	0.5634 (2)	0.0484 (8)	
C20	0.56002 (19)	0.7722 (4)	0.6020 (3)	0.0575 (9)	
H20	0.578193	0.867915	0.574152	0.069*	
C21	0.60862 (18)	0.6788 (4)	0.6820 (3)	0.0536 (8)	
H21	0.659858	0.712093	0.710447	0.064*	

### Atomic displacement parameters (Å^2^)

**Table:**

	*U*^11^	*U*^22^	*U*^33^	*U*^12^	*U*^13^	*U*^23^
O1	0.0689 (15)	0.0619 (14)	0.0268 (11)	0.0189 (11)	0.0064 (10)	−0.0030 (10)
O2	0.0690 (17)	0.0577 (15)	0.0600 (15)	0.0140 (13)	−0.0199 (13)	0.0000 (13)
N1	0.0428 (15)	0.0502 (15)	0.0270 (12)	0.0084 (12)	−0.0001 (11)	0.0012 (11)
N2	0.0424 (14)	0.0476 (15)	0.0232 (12)	0.0101 (12)	−0.0003 (11)	−0.0007 (12)
N3	0.0446 (15)	0.0533 (16)	0.0336 (14)	0.0150 (13)	0.0059 (12)	0.0031 (13)
C1	0.0370 (16)	0.0391 (16)	0.0255 (13)	0.0074 (13)	0.0036 (12)	−0.0007 (12)
C2	0.0424 (17)	0.0420 (17)	0.0295 (15)	0.0038 (14)	0.0031 (13)	0.0027 (13)
C3	0.0336 (16)	0.0436 (17)	0.0320 (15)	0.0014 (13)	0.0035 (13)	0.0018 (13)
C4	0.0423 (17)	0.0398 (16)	0.0367 (16)	0.0017 (14)	0.0091 (14)	−0.0017 (14)
C5	0.052 (2)	0.095 (3)	0.049 (2)	−0.020 (2)	−0.0041 (17)	0.0157 (19)
C6	0.056 (2)	0.126 (4)	0.068 (2)	−0.025 (2)	−0.013 (2)	0.014 (3)
C7	0.045 (2)	0.102 (3)	0.082 (3)	−0.016 (2)	0.011 (2)	−0.011 (3)
C8	0.062 (2)	0.085 (3)	0.082 (3)	−0.019 (2)	0.026 (2)	0.011 (2)
C9	0.056 (2)	0.072 (2)	0.055 (2)	−0.0032 (19)	0.0103 (17)	0.0117 (19)
C10	0.0313 (15)	0.0444 (17)	0.0310 (15)	0.0004 (13)	−0.0014 (12)	0.0018 (13)
C11	0.0445 (18)	0.057 (2)	0.0450 (18)	0.0009 (16)	0.0062 (15)	0.0088 (16)
C12	0.064 (2)	0.072 (3)	0.059 (2)	−0.010 (2)	0.0065 (19)	0.027 (2)
C13	0.062 (2)	0.048 (2)	0.080 (3)	−0.0079 (19)	−0.017 (2)	0.013 (2)
C14	0.061 (2)	0.049 (2)	0.073 (2)	0.0080 (17)	0.0010 (19)	−0.001 (2)
C15	0.0507 (19)	0.049 (2)	0.0496 (19)	0.0065 (15)	0.0081 (16)	0.0004 (16)
C16	0.0404 (18)	0.0432 (18)	0.0381 (16)	0.0096 (14)	0.0038 (14)	0.0025 (14)
C17	0.0455 (19)	0.054 (2)	0.060 (2)	−0.0005 (16)	0.0034 (17)	0.0101 (17)
C18	0.0372 (17)	0.065 (2)	0.061 (2)	0.0037 (17)	−0.0025 (16)	0.0025 (18)
C19	0.0473 (19)	0.049 (2)	0.0432 (17)	0.0110 (16)	−0.0048 (15)	−0.0037 (16)
C20	0.056 (2)	0.0481 (19)	0.063 (2)	−0.0011 (16)	−0.0019 (18)	0.0074 (17)
C21	0.0401 (18)	0.059 (2)	0.056 (2)	0.0008 (17)	−0.0052 (16)	0.0045 (17)

### Geometric parameters (Å, º)

**Table:**

O1—C2	1.230 (3)	C8—H8	0.9500
O2—C19	1.377 (3)	C9—H9	0.9500
O2—H2A	0.859 (12)	C10—C15	1.383 (4)
N1—C3	1.354 (3)	C10—C11	1.390 (4)
N1—C2	1.355 (3)	C11—C12	1.388 (4)
N2—C3	1.337 (3)	C11—H11	0.9500
N2—C1	1.459 (3)	C12—C13	1.369 (5)
N2—H2	0.899 (12)	C12—H12	0.9500
N3—C3	1.329 (3)	C13—C14	1.370 (5)
N3—C16	1.439 (3)	C13—H13	0.9500
N3—H3	0.892 (12)	C14—C15	1.372 (4)
C1—C10	1.515 (4)	C14—H14	0.9500
C1—C4	1.540 (4)	C15—H15	0.9500
C1—C2	1.555 (4)	C16—C17	1.373 (4)
C4—C9	1.373 (4)	C16—C21	1.373 (4)
C4—C5	1.376 (4)	C17—C18	1.385 (4)
C5—C6	1.377 (5)	C17—H17	0.9500
C5—H5	0.9500	C18—C19	1.369 (4)
C6—C7	1.358 (5)	C18—H18	0.9500
C6—H6	0.9500	C19—C20	1.376 (4)
C7—C8	1.352 (5)	C20—C21	1.390 (4)
C7—H7	0.9500	C20—H20	0.9500
C8—C9	1.388 (5)	C21—H21	0.9500
			
C19—O2—H2A	109 (2)	C8—C9—H9	119.6
C3—N1—C2	105.9 (2)	C15—C10—C11	118.1 (3)
C3—N2—C1	109.7 (2)	C15—C10—C1	119.3 (3)
C3—N2—H2	121.4 (18)	C11—C10—C1	122.4 (3)
C1—N2—H2	124.7 (18)	C12—C11—C10	119.9 (3)
C3—N3—C16	124.0 (2)	C12—C11—H11	120.0
C3—N3—H3	117.9 (19)	C10—C11—H11	120.0
C16—N3—H3	117.5 (19)	C13—C12—C11	120.7 (3)
N2—C1—C10	112.8 (2)	C13—C12—H12	119.7
N2—C1—C4	111.0 (2)	C11—C12—H12	119.7
C10—C1—C4	110.6 (2)	C12—C13—C14	119.7 (3)
N2—C1—C2	98.5 (2)	C12—C13—H13	120.2
C10—C1—C2	112.2 (2)	C14—C13—H13	120.2
C4—C1—C2	111.3 (2)	C13—C14—C15	120.0 (3)
O1—C2—N1	125.3 (2)	C13—C14—H14	120.0
O1—C2—C1	123.7 (2)	C15—C14—H14	120.0
N1—C2—C1	111.0 (2)	C14—C15—C10	121.5 (3)
N3—C3—N2	122.1 (3)	C14—C15—H15	119.2
N3—C3—N1	123.3 (2)	C10—C15—H15	119.2
N2—C3—N1	114.6 (3)	C17—C16—C21	119.9 (3)
C9—C4—C5	117.6 (3)	C17—C16—N3	120.4 (3)
C9—C4—C1	122.9 (2)	C21—C16—N3	119.6 (3)
C5—C4—C1	119.5 (3)	C16—C17—C18	120.0 (3)
C4—C5—C6	121.1 (3)	C16—C17—H17	120.0
C4—C5—H5	119.4	C18—C17—H17	120.0
C6—C5—H5	119.4	C19—C18—C17	120.2 (3)
C7—C6—C5	120.5 (3)	C19—C18—H18	119.9
C7—C6—H6	119.7	C17—C18—H18	119.9
C5—C6—H6	119.7	C18—C19—C20	120.2 (3)
C8—C7—C6	119.4 (3)	C18—C19—O2	121.6 (3)
C8—C7—H7	120.3	C20—C19—O2	118.1 (3)
C6—C7—H7	120.3	C19—C20—C21	119.5 (3)
C7—C8—C9	120.6 (4)	C19—C20—H20	120.3
C7—C8—H8	119.7	C21—C20—H20	120.3
C9—C8—H8	119.7	C16—C21—C20	120.2 (3)
C4—C9—C8	120.8 (3)	C16—C21—H21	119.9
C4—C9—H9	119.6	C20—C21—H21	119.9
			
C3—N2—C1—C10	−123.8 (2)	C1—C4—C9—C8	177.0 (3)
C3—N2—C1—C4	111.5 (3)	C7—C8—C9—C4	0.1 (6)
C3—N2—C1—C2	−5.3 (3)	N2—C1—C10—C15	170.7 (2)
C3—N1—C2—O1	179.0 (3)	C4—C1—C10—C15	−64.3 (3)
C3—N1—C2—C1	−1.4 (3)	C2—C1—C10—C15	60.5 (3)
N2—C1—C2—O1	−176.3 (3)	N2—C1—C10—C11	−14.7 (3)
C10—C1—C2—O1	−57.4 (4)	C4—C1—C10—C11	110.3 (3)
C4—C1—C2—O1	67.1 (4)	C2—C1—C10—C11	−124.8 (3)
N2—C1—C2—N1	4.1 (3)	C15—C10—C11—C12	0.1 (4)
C10—C1—C2—N1	123.0 (3)	C1—C10—C11—C12	−174.6 (3)
C4—C1—C2—N1	−112.5 (3)	C10—C11—C12—C13	0.0 (5)
C16—N3—C3—N2	173.9 (3)	C11—C12—C13—C14	−0.3 (5)
C16—N3—C3—N1	−7.2 (5)	C12—C13—C14—C15	0.5 (5)
C1—N2—C3—N3	−175.7 (3)	C13—C14—C15—C10	−0.4 (5)
C1—N2—C3—N1	5.3 (3)	C11—C10—C15—C14	0.1 (4)
C2—N1—C3—N3	178.7 (3)	C1—C10—C15—C14	175.0 (3)
C2—N1—C3—N2	−2.3 (3)	C3—N3—C16—C17	97.0 (4)
N2—C1—C4—C9	−94.0 (3)	C3—N3—C16—C21	−85.6 (4)
C10—C1—C4—C9	140.0 (3)	C21—C16—C17—C18	0.0 (5)
C2—C1—C4—C9	14.6 (4)	N3—C16—C17—C18	177.5 (3)
N2—C1—C4—C5	83.9 (3)	C16—C17—C18—C19	0.6 (5)
C10—C1—C4—C5	−42.0 (4)	C17—C18—C19—C20	−0.2 (5)
C2—C1—C4—C5	−167.4 (3)	C17—C18—C19—O2	−178.4 (3)
C9—C4—C5—C6	1.4 (5)	C18—C19—C20—C21	−0.9 (5)
C1—C4—C5—C6	−176.7 (3)	O2—C19—C20—C21	177.4 (3)
C4—C5—C6—C7	−1.0 (6)	C17—C16—C21—C20	−1.1 (5)
C5—C6—C7—C8	0.1 (6)	N3—C16—C21—C20	−178.6 (3)
C6—C7—C8—C9	0.3 (6)	C19—C20—C21—C16	1.5 (5)
C5—C4—C9—C8	−1.0 (5)		

### Hydrogen-bond geometry (Å, º)

*Cg*3 and *Cg*4 are the centroids of the C10–C15 and the C16–C21 benzene rings, respectively.

**Table:**

*D*—H···*A*	*D*—H	H···*A*	*D*···*A*	*D*—H···*A*
O2—H2*A*···N1^i^	0.86 (1)	1.93 (2)	2.763 (3)	163 (4)
N2—H2···O1^ii^	0.90 (1)	1.92 (1)	2.814 (3)	176 (3)
N3—H3···O2^iii^	0.89 (1)	2.34 (2)	3.104 (4)	143 (2)
C17—H17···*Cg*4^iii^	0.95	2.92	3.831 (4)	162
C21—H21···*Cg*3^iv^	0.95	2.93	3.822 (4)	157

Symmetry codes: (i) −*x*+1, −*y*+1, −*z*+1; (ii) *x*, −*y*+1/2, *z*+1/2; (iii) −*x*+1, *y*−1/2, −*z*+3/2; (iv) *x*, *y*+1, *z*.
